# Efficacy of *Candida dubliniensis* and Fungal β-Glucans in Inducing Trained Innate Immune Protection Against Inducers of Sepsis

**DOI:** 10.3389/fcimb.2022.898030

**Published:** 2022-06-13

**Authors:** Amanda J. Harriett, Shannon Esher Righi, Elizabeth A. Lilly, Paul Fidel, Mairi C. Noverr

**Affiliations:** ^1^ Department of Microbiology and Immunology, Tulane University School of Medicine, New Orleans, LA, United States; ^2^ Center of Excellence in Oral and Craniofacial Biology, Louisiana State University Health Sciences Center School of Dentistry, New Orleans, LA, United States

**Keywords:** trained innate immunity, sepsis, myeloid derived suppressor cell (MDSC), Candida, polymicrobial intra-abdominal infection, zymosan, lipopolysaccharide (LPS)

## Abstract

Fungal-bacterial intra-abdominal infections (IAI) can lead to sepsis with significant morbidity and mortality. We have established a murine model of *Candida albicans* (*Ca*) and *Staphylococcus aureus* (*Sa*) IAI that results in acute lethal sepsis. Prior intraperitoneal or intravenous inoculation with low virulence *Candida dubliniensis (Cd)* confers high level protection against lethal *Ca/Sa* IAI and sepsis. Protection *via Cd* immunization is associated with decreased pro-inflammatory cytokines and mediated by Gr-1^+^ putative myeloid-derived suppressor cells (MDSCs) representing a novel form of trained innate immunity (TII). The objective of these studies was to determine the extent of *Cd*-mediated TII against sepsis of broad origin and explore the potential of fungal cell wall components as abiotic immunogen alternatives to induce TII, including zymosan depleted of TLR2 activity (d-zymosan), or purified preparations of β-glucan. Immunized mice were challenged 14 days post-immunization with a lethal array of live or abiotic inducers of sepsis, including *Ca/Sa, Ca*/*Escherichia coli* (*Ca/Ec*), LPS or untreated zymosan. Results showed that live *Cd* immunization was protective against sepsis induced by *Ca/Ec* and zymosan, but not LPS. Similar to protection against Ca/Sa, survival was dependent on Gr-1+ cells with no role for macrophages. Among the fungal cell wall compounds as immunogens, immunization with d-zymosan and an alkali-treated form of β-glucan also resulted in significant protection against sepsis induced by *Ca/Sa* or *Ca/Ec*, but not LPS sepsis. Again, there was a strong dependence on Gr-1+ cells for protection with one exception, an added role for macrophages in the case of protection induced by alkali-treated β-glucan. Overall, these results demonstrate that immunization with *Cd* as well as abiotic fungal cell components are capable of Gr-1+ cell-mediated trained innate immune protection against sepsis of broad microbial origin. In addition, abiotic β-glucans represent potential alternatives to live *Cd* for protection against lethal polymicrobial sepsis.

## Introduction

Sepsis, a major cause of morbidity and mortality in critically ill patients, is the result of unrestrained inflammatory immune responses to pathogens that can result in organ dysfunction and failure, circulatory disruption, and eventual death. According to the CDC, at least 1.7 million adults in the U.S. develop sepsis annually and one of every three patient deaths in a hospital involves sepsis (Rhee et al., 2017). Despite its clinical importance, there are no vaccines available that target sepsis and few effective treatments or options for supportive care (Gyawali et al., 2019). The key to controlling sepsis lies in the prevention and/or suppression of uncontrolled host inflammation. Various animal models of sepsis have been developed to study the immunopathogenesis of disease and include models that use abiotic microbial toxins or PAMPs (pathogen associated molecular patterns) or live microbes inoculated systemically or *via* disruption in mucosal barriers such as the GI tract (e.g., cecal-ligation puncture) (van der Poll, 2012; Lewis et al., 2016). Lethal sepsis is a common sequela of GI perforations leading to intra-abdominal infections (IAI) if left untreated or misdiagnosed (Muresan et al., 2018; Blot et al., 2019). Our laboratory has been studying fungal/bacterial sepsis using an experimental mouse model of *C. albicans/S. aureus (Ca/Sa)* polymicrobial IAI which results in 80-90% mortality by 48 to 72 h post-inoculation, which is associated with significantly enhanced systemic proinflammatory cytokines rather than microbial burden (Peters and Noverr, 2013; Nash et al., 2014; Nash et al., 2015).

Use of non-*albicans Candida* species with *S. aureus* in the lethal intraperitoneal (i.p.) challenge results in varying levels of mortality. Of these, co-infection with *C. dubliniensis* (*Cd;* a close phylogenetic relative of *C. albicans*) and *S. aureus* resulted in minimal mortality (~10-20%). Interestingly, surviving mice given a lethal challenge with *Ca/Sa* 14 days later resulted in extremely high survival (80-90%) (Nash et al., 2015; Lilly et al., 2018). Subsequent studies demonstrated that animals inoculated with *C. dubliniensis* alone, as a monomicrobial primary challenge (immunization), were equally protected (80-90%) against lethal *Ca/Sa* IAI. This protection was long-lived (up to 60 days post-*Cd* immunization), but not mediated by adaptive immunity (protection was maintained in RAG1^-/-^ mice lacking T and B cells) (Lilly et al., 2018). This suggested that protection was mediated by innate cells potentially *via* trained innate immunity (TII), which is defined as enhanced responsiveness by innate cells such as macrophages and NK cells in response to a secondary challenge (Netea et al., 2011). However, clodronate-mediated depletion of phagocytic macrophages failed to abrogate protection (Lilly et al., 2018). Instead, protection was abrogated following antibody depletion of Ly6G^+^ Gr-1^+^ leukocytes indicated a novel role for polymorphonuclear neutrophils (PMNs) in mediating protection (Lilly et al., 2018; Lilly et al., 2021). With protection lasting for at least 60 days post-vaccination, and considering the short lifespan (24 h) of PMNs, these results suggested that the protective Gr-1^+^ cells were putative, long-lived myeloid-derived suppressor cells (MDSCs) which have been reported in other models of sepsis (Schrijver et al., 2019) and in patients with candidiasis (Rieber et al., 2015).

Our laboratory subsequently proposed the concept that Gr-1^+^ MDSCs with potent anti-inflammatory activity mediate a novel form of trained innate immunity against sepsis, termed trained tolerogenic immunity (TTI) (Esher et al., 2019). TTI is likely a part of a spectrum of trained innate responses that are not mutually exclusive, including pro-inflammatory/anti-microbial and tolerogenic responses, which vary depending on the cellular induction and timing/route of administration. Immunization with live attenuated vaccines (LAV) has been historically noted to induce beneficial non-specific effects (NSE) against unrelated infections, a phenomenon termed heterologous immunity. Several studies have reported significantly reduced mortality from unrelated respiratory infections and neonatal sepsis in pediatric populations immunized with common live vaccines including BCG, measles or smallpox vaccines (Aaby and Benn, 2019). It was proposed that the NSE reported for LAV were at least in part attributable to innate cellular training, which involves epigenetic reprogramming induced by alterations in intracellular metabolic pathways (Cheng et al., 2014; Blok et al., 2015; Kleinnijenhuis et al., 2015; Arts et al., 2016). TII is often triggered by microbial PAMPs (pathogen associated molecular patterns) and PRR (pattern recognition receptor) signaling; therefore, strategies to develop and optimize abiotic inducers of TII are being actively pursued as alternatives to or adjuvants for live vaccines (Sanchez-Ramon et al., 2018). Among abiotic inducers, β-glucan, a fungal cell wall component, has been demonstrated to prime monocytes for enhanced pro-inflammatory cytokine production and improved antimicrobial activity by inducing a unique epigenetic signature (Quintin et al., 2012; Saeed et al., 2014). The goal of these studies was to evaluate the efficacy of protection induced by immunization with live *Cd* vs. abiotic fungal β-glucan compounds in various experimental models of sepsis induced by live microbes or abiotic microbial stimuli and to determine the relative importance of Gr-1+ leukocytes vs. monocyte/macrophage populations in mediating protection.

## Materials and Methods

### Mice

For all experiments, female Swiss Webster mice, 6 to 8 weeks of age, were purchased from Charles River Laboratories, Inc. Animals were housed and handled according to institutionally recommended guidelines. Mice that reached clinical endpoints prior to study endpoint were humanely euthanized following IACUC-approved euthanasia procedures. All experiments involving animals were approved by the Tulane Institutional Animal Care and Use Committee.

### Microbial Strains and Growth Conditions

*C. albicans* strain DAY185, a prototrophic derivative of SC5314, was a gift from Aaron Mitchell (Carnegie Mellon University, Pittsburgh, PA). The *C. dubliniensis* wild-type strain (Wü284) was kindly provided by Gary Moran (Trinity College, Dublin, Ireland). Frozen stocks were maintained at −80°C and streaked onto yeast extract-peptone-dextrose (YPD) agar prior to use. A single colony was transferred to 10 ml of YPD broth, and the culture was shaken at 30°C for 12 to 18 h. The methicillin-resistant *S. aureus* strain NRS383 used in all experiments was obtained from the Network on Antimicrobial Resistance in *Staphylococcus aureus* (NARSA) data bank. Frozen stocks were maintained at −80°C and streaked onto Trypticase soy agar (TSA) prior to use. The *E. coli* strain 25922 (ATCC) used in all experiments, a gift from Jacob Bitoun (Tulane University School of Medicine, New Orleans, LA), was chosen based on previous publications documenting sepsis or peritonitis models (Cirioni et al., 2006; Chen et al., 2010; Ghiselli et al., 2011). The strain ATCC 25922 is a commonly used quality control strain, particularly in antibody sensitivity assays and was originally isolated from a human clinical sample collected in Seattle and WA (1946). It is of serotype O6 and biotype 1.

Frozen stocks were maintained at −80°C and streaked onto Trypticase soy agar (TSA) prior to use. For both bacterial pathogens, a single colony was transferred to 10 ml of Trypticase soy broth (TSB) or Luria Broth (LB) and shaken at 37°C overnight. On the following day, the overnight culture was diluted 1:100 in fresh TSB/LB and shaken at 37°C for 1.5-3 h until the culture reached the log phase of growth. Prior to inoculation, cultures of both organisms were washed 3 times by centrifugation in sterile PBS (pH 7.4), counted using a hemocytometer, and diluted in sterile PBS to prepare standardized inocula.

### Immunizations

For all immunization experiments, groups (n = 4-10) of 6-week-old outbred Swiss Webster or inbred C57BL/6J were used.

#### Live *C. dubliniensis* (*Cd*)

Mice were inoculated i.p. with 200 ul *Cd* (1.75 × 10^7^/mouse) resuspended in sterile non-pyrogenic PBS 14 d prior to sepsis challenge.

#### Abiotic Fungal Cell Wall Compounds

Mice were injected i.p. with various β-glucan preparations prior to challenge in sepsis models. Purified β-glucan from *S. cerevisiae* (Millipore, cat# 346210): Mice were injected with 1 dose of 1-4 mg untreated or modified β-glucan 7-14 days prior to sepsis challenge. To produce modified β-glucan, purified β-glucan was alkali treated by overnight incubation at room temperature in 0.1 M borate buffer (pH = 9.8). The solution was centrifuged, and pellet washed 3 times in sterile PBS, and resuspended in 0.2 N NaOH and incubated for 20 min at room temperature. The solution was centrifuged, and pellet washed 3 times with sterile DI H_2_O and resuspended in sterile non-pyrogenic PBS prior to injection.

Depleted Zymosan from *S. cerevisiae* (zymosan-D; *In vivo*gen, cat# tlrl-zyd): Mice were injected with 1 (day -14) or 2 (day -14, day -7) doses of 1.2 mg zymosan-D dissolved in sterile non-pyrogenic PBS prior to sepsis challenge.

Whole glucan particulate (WGP dispersible; *In vivo*gen, cat#tlrl-wgp): Mice were injected with 1 dose of 200μg WGP dissolved in sterile non-pyrogenic PBS 14 days prior to sepsis challenge. The concentrations of β-glucans (molecular and WGP dispersible) were based on studies by Moorlag et al. where *in vivo* trained innate immune responses were elicited in mice who had been immunized intraperitoneally with 1 mg β-glucan (Moorlag S.J.C.F. et al., 2020). Increased doses were tested to optimize protective effects. Highest efficacy against polymicrobial challenge was observed using one dose of 4 mg β-glucan.

### Murine Models of Sepsis

Polymicrobial IAI: Mice were injected i.p. with a lethal challenge of *C. albicans* (1.75 × 10^7^/mouse) and *E. coli* (4.5 × 10^6^/mouse) in a volume of 200 µl. In both aseptic and polymicrobial rechallenge models the mice were observed for morbidity using the modified M-CASS (scores consider a range from 0-3 when observing aspects such as fur ruffling, activity level, posture/hunching, behavior, respiration quality/rate and squinting or orbital tightening) and mortality up to 10 days after challenge (Mai et al., 2018). Mice who reached clinical endpoints prior to study endpoint were humanely euthanized following IACUC-approved euthanasia procedures. To assess microbial burdens, spleens (homogenized in 500 μl sterile PBS) and 1 ml peritoneal lavage fluid were collected from each mouse at clinical or study endpoint. CFUs were enumerated by plating serial dilutions of spleen homogenates or peritoneal lavage samples onto YPD agar containing 40 μg/ml gentamycin and 2 μg/ml vancomycin for fungal colonies and TSA agar containing 40 µg/ml gentamycin and 2.5 µg/ml amphotericin B for bacterial colonies. Plates were incubated overnight at 37°C. CFU counts are expressed as the number of CFU/ml of peritoneal lavage fluid and CFU/g of spleen homogenates.

Zymosan: Mice were injected i.p. with a lethal challenge of 700-1000 mg/kg of zymosan obtained from *S. cerevisiae* (Sigma Aldrich) and resuspended in sterile NaCl.

LPS: Mice were injected i.p. with a lethal challenge of 300 μg (10 mg/kg) of LPS obtained from *E. coli* O111:B4 (Sigma Aldrich) and resuspended in sterile NaCl.

### Cell Depletion

For macrophage depletion, liposome-encapsulated clodronate and liposome vehicle (1 mg/mouse; Encapsula NanoSciences) were injected i.p. in 200 µl 1 day prior to sepsis challenge. Clodronate (dichloromethylene-bisphosphonate) is encapsulated in the aqueous compartments of liposomes which have been filtered for size to remove larger particles that might be toxic to animals. The liposomal solution is injected intraperitoneally into the mice where phagocytic cells recognize the liposomes as foreign particles and phagocytose them. When internalized, the liposomes release clodronate into the cytosol, resulting in cell death. Liposomes without clodronate exhibit no cellular toxicity.

For granulocyte depletion, mice were injected i.p. with either 200 μg rat anti-mouse Gr-1^+^ (Ly6G/Ly6C) or rat IgG2A isotype control antibodies (Bio-X-Cell) in 200 µl sterile non-pyrogenic PBS to systemically deplete PMNLs 48 h prior to and 2 h after challenge. Injections were given every 2 days for the duration of the study. Depletion was confirmed by flow cytometry.

### Sepsis Scoring

Mice are monitored for survival in three checks per day following challenge for 10 days. Daily behavioral scoring is performed using a modified sepsis scoring criteria including fur aspect, activity level, posture, breathing quality and grimace signs to quantify morbidity and predict mortality in mice (Mai et al., 2018). Onset of sepsis is rapid in unvaccinated mice; therefore, scoring in these groups is monitored for all mice through study or clinical endpoint but only reported up to the time that the majority of animals in the group have been euthanized to eliminate presentation of results that are not representative of the group as a whole.

### Statistics

Survival curves were compared using the log rank (Mantel-Cox) test. Significant differences were defined at *P*<0.05. Sepsis scores were compared using ANOVA followed by *post hoc* Student’s t-test. For microbial burden assessments, calculated CFU counts were log transformed for the purpose of normalization and analyzed using a one-way ANOVA with Tukey’s test for multiple comparisons. These statistical analyses were performed using Prism software (Graph Pad Prism 9).

## Results

### *C. dubliniensis* Induces Gr-1+ Cell-Mediated Protection Against Lethal Challenge With *C. albicans/E. coli* But Not LPS

**Figure 1:**
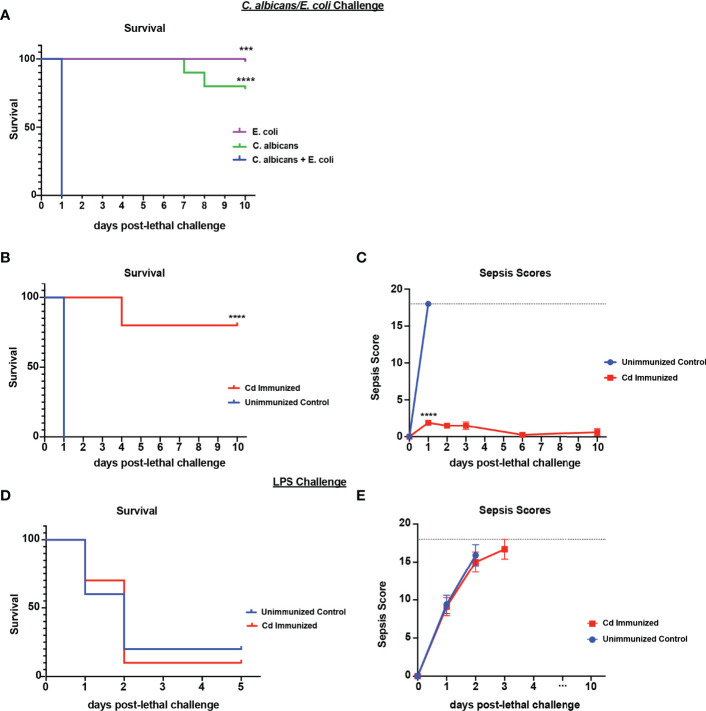
Immunization with *Candida dubliniensis* induces protection against polymicrobial *C. albicans*/*E. coli* IAI but not LPS-induced sepsis. Initial studies confirmed that polymicrobial challenge of *C. albicans* (1.75x10^7^ CFUs) and *E. coli* (4.5x10^6^ CFUs) was lethal whereas monomicrobial challenge was not lethal (n=10 mice/group) (representative results of six repeats shown in **A**). For protection studies, mice (n = 10/group) were given 1.75x10^7^ CFUs of live *C. dubliniensis* (strain Wü284), i.p. as an immunization followed by challenge with lethal *C. albicans* (1.75x10^7^ CFUs) and *E. coli* (4.5x10^6^ CFUs) **(B, C)** or lethal LPS (10 mg/kg) **(D, E)**, i.p. and monitored for survival and morbidity/sepsis scoring following lethal challenge for 10 days. Animals receiving no primary challenge served as the negative (lethal) control. Mice were monitored and scored for 10 days post-lethal challenge. Graphs are representative of at least 3 separate experiments. *, P < 0.05; **, P < 0.01; ***, P < 0.001; ****, P < 0.0001 [for values significantly different from those of the control, by log rank Mantel-Cox test (survival) and ANOVA followed by *post hoc* Student’s t-test (sepsis scoring)].

We have previously established that immunization with live *Cd* 14-60 days prior to lethal challenge effectively induces trained innate protection against sepsis induced by polymicrobial IAI with several pathogenic *Candida* species *and S. aureus* (Lilly et al., 2018). Importantly, *Cd* is largely cleared by day 7 post-immunization with no evidence of further burden prior to challenge (Lilly et al., 2021). To determine whether this protection extends to IAI involving a common gram-negative bacterial abdominal pathogen, we first gave mice a lethal challenge of *C. albicans* and *Escherichia coli* strain ATCC 25922 (*Ca*/*Ec*). Similar to our murine model of *Candida sp/Sa* IAI, intraperitoneal inoculation of *Ca/Ec* results in synergistic lethality (Klaerner et al., 1997), whereas monomicrobial infections of each failed to result in mortality (**Figure 1A**). To evaluate protection, mice were inoculated (immunized) i.p. with *Cd* and challenged 14 d later by i.p. inoculation with *Ca/Ec.* Mice were monitored for survival and sepsis scores using the modified M-CASS system (Mai et al., 2018) over a 10-day period. Results showed that prior immunization of mice with *Cd* resulted in 80% survival following *Ca/Ec* challenge compared with no survival in unvaccinated mice (p<0.0001) (**Figure 1B**). Unvaccinated mice exhibited severe morbidity including ruffling of fur, squinting and hunched posture prior to humane euthanasia indicated by high sepsis scores in 24-48 h, while immunized mice exhibited low sepsis scores with a limited number of sepsis/morbidity indicators over the 10-day period (**Figure 1C**). We next examined the efficacy of the live *Cd* immunization in protecting against sepsis induced by *E. coli*-derived LPS (LPS endotoxin serotype O111:B4) (10 mg/kg). *Cd*-immunized mice challenged 14 d later by i.p. injection with a lethal dose of LPS rapidly succumbed to sepsis, with 10% survival by 48 h post-injection which was not significantly different from the 20% survival observed in unvaccinated mice (**Figure 1D**) with high sepsis scoring in both groups (**Figure 1E**).

**Figure 2:**
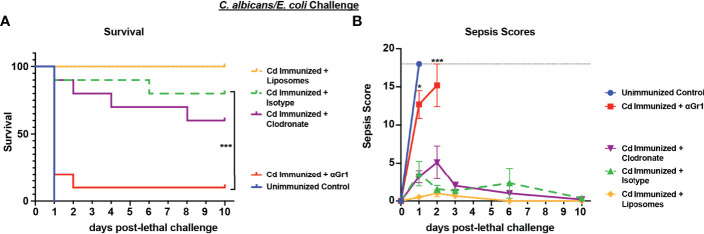
Role of macrophages and Gr-1+ leukocytes in *C. dubliniensis*-induced protection against polymicrobial *C. albicans*/*E. coli* IAI. Mice (n = 10/group) were immunized by i.p. injection with 1.75x10^7^ CFUs of live *C. dubliniensis* 14 days prior to lethal challenge. For macrophage depletion, mice were injected i.p. with liposome-encapsulated clodronate or control empty liposomes 1 day prior to lethal challenge. For Gr-1+ cell depletion, mice were injected i.p. with 200 μg anti-Gr-1 or isotype control antibody 48 h prior to and 2 h after lethal challenge. Mice were challenged with *C. albicans* (1.75x10^7^ CFUs) and *E. coli* (4.5x10^6^ CFUs) i.p. and monitored for **(A)** survival and **(B)** morbidity/sepsis scoring for 10 days post-lethal challenge. Naïve (unvaccinated) mice served as the negative control. Graphs are representative of 2 separate experiments. *, P < 0.05; **, P < 0.01; ***, P < 0.001; ****, P < 0.0001 [for values significantly different from those of the control, by log rank Mantel-Cox test (survival) and ANOVA followed by *post hoc* Student’s t-test (sepsis scoring)].

Based on the strong protection imparted by live *Cd* immunization against *Ca/Ec* challenge, we next tested the requirement for innate immune cell populations previously identified in other models (Gr-1+ leukocytes or macrophages) in mediating trained immune protection (Quintin et al., 2012; Lilly et al., 2018). For this, *Cd* immunized mice were treated with anti-Gr-1^+^ or isotype control antibodies every 48h beginning 48h prior to lethal challenge, or a single injection of clodronate-containing liposomes or empty liposomes 24h prior to lethal challenge with *Ca/Ec*, and monitored for mortality and sepsis scoring. Compared to the 80-100% survival in *Cd* immunized untreated or isotype antibody-treated control mice, Gr-1^+^ (Ly6C/Ly6G) cell depleted mice had high mortality rates (p<0.001) equivalent to unvaccinated control mice (**Figure 2**). In contrast, clodronate-treated mice depleted of macrophages had survival rates similar to the empty liposome-treated mice and significantly higher than the unvaccinated control mice (p< 0.001) (**Figure 2A**). Sepsis scores paralleled the mortality/survival results (**Figure 2B**), including scores reflecting the modest reduction in survival in macrophage-depleted mice (~60%) (~5 at day 2 post-challenge) compared with control liposome treated mice (~1).

### *C. dubliniensis* Immunization Protects Against Zymosan Induced Sepsis With a Similar Role for Gr-1+ Cells in Protection

**Figure 3:**
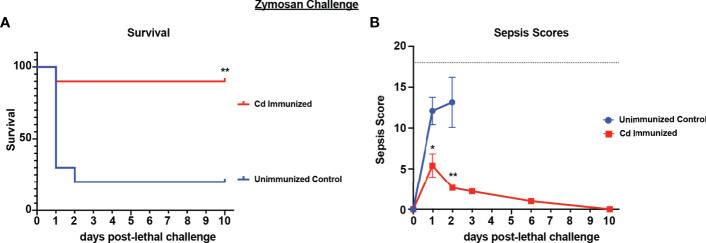
Immunization with *Candida dubliniensis* induces protection against zymosan-induced sepsis. Mice (n = 10/group) were given 1.75x10^7^ CFUs of live *C. dubliniensis* (strain Wü284), i.p. as an immunization followed by challenge with lethal 700-1000 mg/kg zymosan and monitored for **(A)** survival and **(B)** morbidity following lethal challenge for 10 days. Animals receiving no primary challenge served as the negative (lethal) control. Mice were monitored for survival and morbidity/sepsis scoring for 10 days post-lethal challenge. Naïve (unvaccinated) mice served as the negative control. Graphs are representative of 3 separate experiments. *, P < 0.05; **, P < 0.01; ***, P < 0.001; ****, P < 0.0001 [for values significantly different from those of the unvaccinated control, by log rank Mantel-Cox (survival) and ANOVA followed by *post hoc* Student’s t-test (sepsis scoring)].

Because live *Cd* immunization induced protection in several models of fungal/bacterial IAI, we tested whether the protection extends to sepsis induced by zymosan, a cell wall extract of yeast that mimics human sepsis when administered i.p. at high concentrations by signaling *via* both TLR-2 and dectin-1 (Dillon et al., 2006; Lewis et al., 2016; Stortz et al., 2017). For these studies, mice were immunized with live *Cd* 14 d prior to i.p. challenge with a lethal dose of zymosan (1000mg/kg) and monitored for mortality and sepsis scores. Results showed that by day 3 post-challenge, the majority of unvaccinated mice had succumbed to sepsis (~20% survival). In contrast, the majority of immunized mice survived through day 10 (~90% survival; p<0.01) (**Figure 3A**). Correspondingly, immunized mice exhibited mild signs of sepsis over the 10 day period, while unvaccinated mice displayed severe morbidity resulting in high sepsis scores within 24 h of lethal challenge (**Figure 3B**).

**Figure 4:**
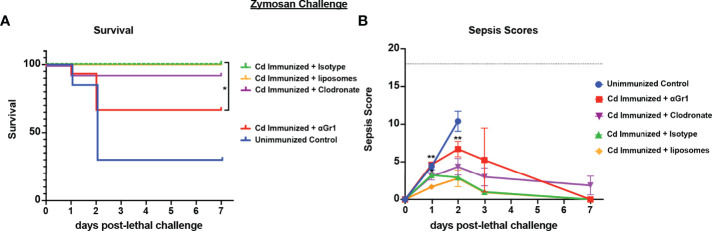
Role of macrophages and Gr-1+ leukocytes in *C. dubliniensis*-induced protection in zymosan-induced sepsis. Mice (n = 10/group) were immunized by i.p. injection with 1.75x10^7^ CFUs of live *C. dubliniensis* 14 days prior to lethal challenge. For macrophage depletion, mice were injected i.p. with liposome-encapsulated clodronate or control empty liposomes 1 day prior to lethal challenge. For Gr-1+ cell depletion, mice were injected i.p. with 200 μg anti-Gr-1 or isotype control antibody 48 h prior to and 2 h after lethal challenge. Mice were challenged with 700-1000 mg/kg zymosan and monitored for **(A)** survival and **(B)** morbidity/sepsis scoring following lethal challenge for 10 days. Naïve (unvaccinated) mice served as the negative control. Graphs are cumulative data from 3 separate experiments. *, P < 0.05; **, P < 0.01; ***, P < 0.001; ****, P < 0.0001 [for values significantly different from those of the control, by log rank Mantel-Cox test (survival) and ANOVA followed by *post hoc* Student’s t-test (sepsis scoring)].

To determine whether the cell populations necessary for effective *Cd* protection were the same as previously identified in both polymicrobial infection models, we repeated the challenge model in mice depleted of either macrophages or Gr-1^+^ cells using the same design. Results showed significantly reduced survival in immunized Gr-1+ cell depleted mice (60% survival) compared to the isotype antibody control immunized mice (100% survival) (p<0.05). Despite the uncharacteristically higher survival in Gr-1+ cell depleted mice, there was no statistical significance compared to unvaccinated mice (~40% survival). In contrast, survival in clodronate-treated and empty-liposome-treated immunized mice were similarly high (90% and 100%, respectively) (**Figure 4A**). These effects are consistent with the sepsis scoring with Gr-1+ cell-depleted vaccinated mice having moderate-high scores similar to those of the unvaccinated mice (7-10 by day 2 post challenge), and mild sepsis scores (<5) in both groups of control-treated and clodronate-treated immunized mice throughout the observation period (**Figure 4B**).

### Immunization With Fungal β-Glucan Compounds Results in Protection Against Polymicrobial Ca/Sa IAI Sepsis Predominantly Mediated by Gr-1+ Cells

**Figure 5:**
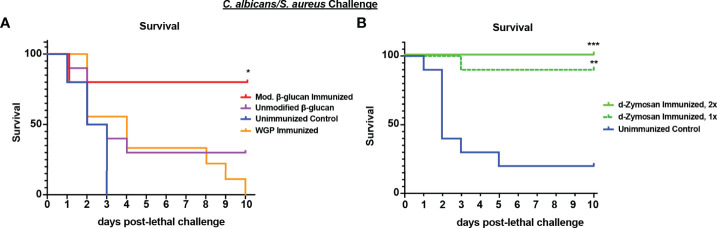
Immunization with β-glucan preparations induces protection against lethal polymicrobial *C. albicans/S. aureus* IAI. **(A)** Mice (n = 10/group) were immunized by i.p. injection with unmodified (1 mg), modified β-glucan (1mg), or whole glucan particle dispersible (WGP, 200 μg) 14 days prior to challenge. **(B)** Mice (n=10/group) were immunized by i.p. injection with 1 dose (day -14) or 2 doses (day -14, -7) of d-zymosan (1.2 mg) prior to challenge. For lethal IAI challenge, mice were inoculated by i.p. injection with *C. abicans* (1.75x10^7^ CFUs) and *S. aureus* (8x10^7^ CFUs) and monitored for survival and morbidity/sepsis scoring for 10 days. Naïve (unvaccinated) mice served as the negative control. Graphs are representative of 2-3 separate experiments. *, P < 0.05; **, P < 0.01; ***, P < 0.001; ****, P < 0.0001 (for values significantly different from those of the control, by log rank Mantel-Cox test).

Previously published studies have identified abiotic agents as inducers of TII including fungal β-glucan, a component of the fungal cell wall (Quintin et al., 2014). We tested a variety of commercially available fungal β-glucan compounds for efficacy in inducing protection against polymicrobial IAI induced sepsis. Different formulations of fungal β-glucans are available as both soluble and particulate compounds that differ in receptor signaling capacity. For these studies, several preparations of purified β-glucan derived from *S. cerevisiae* were tested. The particulate β-glucan was either treated by incubation in alkaline buffer to increase solubility (modified) or left untreated and resuspended in sterile PBS (unmodified). We also tested a larger size β-glucan product, WGP (whole glucan particle) dispersible, which consists of hollow whole *S. cerevisiae* cell wall “ghosts”, which function as a dectin-1 agonist (Goodridge et al., 2011). Mice were immunized by i.p. injection with 1 mg of the β-glucan preparations or 200 ug WGP dispersible and challenged 14 d later with lethal *Ca/Sa* IAI. While unmodified β-glucan and WGP were ineffective in inducing protection (~30% survival at day 4 post-challenge vs 0% survival in unvaccinated mice), modified (alkali-treated) β-glucan induced strong protection with 80% survival at day 10 post-challenge (p<0.05) (**Figure 5A**). Even at higher doses, the untreated purified β-glucan showed no appreciable protection (data not shown). Lastly, we tested *S. cerevisiae* depleted zymosan (d-zymosan), which is treated with hot alkali solution to remove TLR signaling activity while retaining dectin-1 signaling (Ikeda et al., 2008). Mice were immunized by i.p. injection with 1 (day -14) or 2 (day -14, -7) doses of 1.2 mg d-zymosan and challenged with *Ca/Sa* IAI. Similar to our results with modified β-glucan, immunization with 1 or 2 doses of d-zymosan induced significant protection against sepsis with 90-100% of mice surviving through day 10 post-lethal challenge compared to 20% survival in unvaccinated mice (p<0.001) (**Figure 5B**).

**Figure 6:**
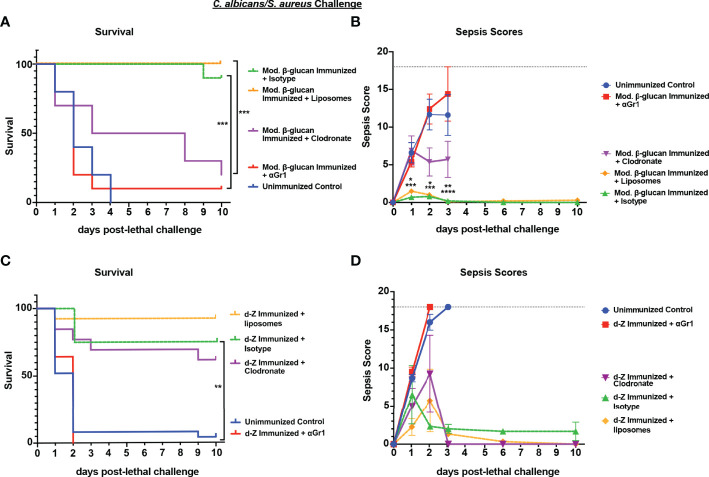
Role of macrophages and Gr-1+ leukocytes in β-glucan-induced protection against lethal polymicrobial *C. albicans/S. aureus* IAI. Mice (n= 10/group) were immunized with either modified β-glucan **(A, B)** or d-zymosan **(C, D)** prior to lethal challenge as described in **Figure 5**. For macrophage depletion, mice were injected i.p. with liposome-encapsulated clodronate or control empty liposomes 1 day prior to lethal challenge. For Gr-1+ cell depletion, mice were injected i.p. with 200 μg anti-Gr-1 or isotype control antibody 48 h prior to and 2 h after lethal challenge. Mice were challenged with 700-1000 mg/kg zymosan and monitored for **(A, C)** survival and **(B, D)** morbidity/sepsis scoring following lethal challenge for 10 days. Naïve (unvaccinated) mice served as the negative control. Graphs are representative of at least 3 separate experiments. The exception is **(A)** that shows cumulative data of 3 separate experiments. *, P < 0.05; **, P < 0.01; ***, P < 0.001; ****, P < 0.0001 [for values significantly different from those of the control, by log rank Mantel-Cox test (survival) and ANOVA followed by *post hoc* Student’s t-test (sepsis scoring)].

To determine whether the protection against IAI sepsis induced by abiotic β-glucan compounds was mediated by the same cell population that we had identified previously in *Cd* vaccination (i.e., Gr-1^+^ MDSCs), we performed cell depletions as described above and monitored survival and sepsis scores. Following immunization with modified β-glucan, we observed significant mortality in Gr-1+ cell depleted mice (10% survival) compared with isotype antibody-treated control mice (90% survival) (p<0.001) and delayed mortality in immunized macrophage-depleted mice (**Figure 6A**) (p<0.001) compared to immunized control liposome-treated mice. Survival in the macrophage-depleted mice was significantly higher compared with unvaccinated mice (p<0.05) but not significantly different from survival in Gr-1^+^-depleted mice. These patterns are reflected in the results of sepsis scoring with both the Gr-1^+^-depleted mice and unvaccinated mice having high scores (range 10-18) by day 2 post-lethal challenge, and macrophage-depleted mice with modest scores (range 3-6) by day 3 post-lethal challenge (**Figure 6B**). In contrast, the effects of cellular depletions in mice immunized with d-zymosan were more similar to *Cd* immunized mice; Gr-1+ cell depletion completely abrogated protection compared with isotype antibody control mice (0% vs 80% survival, respectively), while survival in macrophage-depleted mice was similar to that for empty liposome control mice (60% vs 90% survival, respectively) (**Figure 6C**). Sepsis scores followed the same trends as mortality with unvaccinated and Gr-1^+^-depleted immunized mice having high sepsis scores compared to the vaccinated control mice, and macrophage-depleted immunized mice having overall scores similar to vaccinated mice (**Figure 6D**). To determine whether protection induced by abiotic immunization resulted in reduced microbial burdens similar to previous studies (Lilly et al., 2021), peritoneal lavage fluid and spleens were analyzed for CFU levels at clinical endpoint for moribund mice or study endpoint for healthy animals. Immunization with either d-zymosan or alkali-treated β-glucan resulted in clearance of both *C. albicans* and *S. aureus* in most tissues at study endpoint compared with high levels in unvaccinated animals at clinical endpoint (**Figure S1**).

### Fungal β-Glucan Compounds Induce Protection Against Polymicrobial Ca/Ec IAI But Not LPS Sepsis

**Figure 7:**
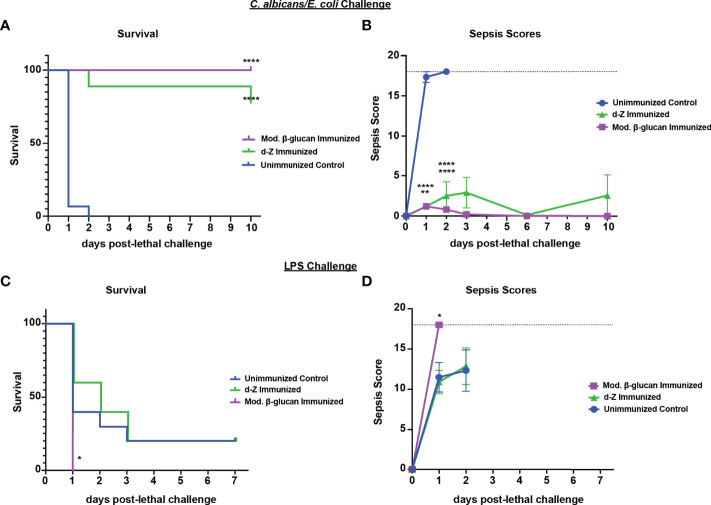
Immunization with β-glucan preparations induces protection against lethal polymicrobial *C. albicans/E.coli* IAI but not LPS-induced sepsis. Mice (n= 10/group) were immunized with either modified β-glucan or d-zymosan prior to lethal challenge as described in **Figure 5**. Mice were then challenged by i.p. injection with *C. albicans* (1.75x10^7^ CFUs) and *E. coli* (4.5x10^6^ CFUs) **(A, B)** or LPS (10 mg/kg) **(C, D)** and monitored for survival **(A, C)** and morbidity/sepsis scoring **(B, D)** for 7-10 days post-lethal challenge. Naïve (unvaccinated) mice served as the negative control. Graphs are representative of 2-4 separate experiments and (A&B) a cumulative of 2 separate experiments. *, P < 0.05; **, P < 0.01; ***, P < 0.001; ****, P < 0.0001 [for values significantly different from those of the control, by log rank Mantel-Cox test (survival) and ANOVA followed by *post hoc* Student’s t-test (sepsis scoring)].

To determine if protection induced by abiotic β-glucan compounds against *Ca/Sa* sepsis also extends to the other models of sepsis, we tested their efficacy against *Ca/Ec* IAI and LPS-induced sepsis. For these experiments, mice were immunized once with modified β-glucan or twice with d-zymosan followed by lethal challenge of *Ca/Ec* i.p. or LPS 14 days later and monitored for survival and sepsis scoring. Similar to immunization with live *Cd*, both β-glucan compounds induced strong protection (75-100% survival through day 10) compared with unvaccinated mice (p<0.0001) (**Figure 7A**). The protection in the immunized mice corresponded with low sepsis scores compared to unvaccinated mice (**Figure 7B**) (p<0.0001). Similar to immunization with live *Cd* (**Figure 1**), both β-glucan compounds failed to confer any significant protection against the LPS challenge (**Figure 7C**). This was corroborated with the high sepsis scores in both vaccinated and unvaccinated mice (**Figure 7D**).

## Discussion

These studies significantly extend our previous reports of TTI-mediated protection against polymicrobial sepsis (Lilly et al., 2017; Lilly et al., 2018; Lilly et al., 2019; Lilly et al., 2021) by demonstrating that immunization with live *Cd* or abiotic fungal compounds confers protection against polymicrobial sepsis of broad microbial origin, including both Gram^-^ and Gram^+^ bacterial pathogens, as well as abiotic inducers of sepsis. The significance of this fungal-induced TTI protection against polymicrobial IAI of broad microbial origin is enhanced when considering the increased lethality reported for polymicrobial infections that include *Candida* (upwards of 75% mortality) (Alden et al., 1989; Calandra et al., 1989; Montravers et al., 1996; Dupont et al., 2002; Sandven et al., 2002; Hughes et al., 2005; Montravers et al., 2006; Miles et al., 2009). While *S. aureus* and *E. coli* are two important causative agents in polymicrobial IAI, we recognize that these species are only one representative of Gram^+^ and Gram^-^ bacterial pathogens. However, we predict that the TTI protection will be broadly effective against a wide array of infections that cause sepsis. This is supported by the fact that TTI induced by *Candida* also protects against fungal sepsis induced by bloodstream infection **(** Lilly et al., 2019 **)**.

*E. coli* is a ubiquitous enteric bacterial pathogen and is often co-isolated along with *C. albicans* in abdominal infections **(** Vergidis et al., 2016 **)**, causing synergistic mortality in experimental models of IAI during co-infection with *C. albicans* **(** Klaerner et al., 1997 **)**. The studies presented here also confirm the synergistic effect of *E. coli* and *C. albicans* on mortality. While monomicrobial *E. coli* infections failed to induce any appreciable morbidity or lethal sepsis, co-infection resulted in acute sepsis and ~100% mortality by 48h post-infection. These results are similar to what we and others previously reported for polymicrobial IAI with *C. albicans* and *S. aureus* **(** Carlson, 1982 **;** Peters and Noverr, 2013 **)**. Another significant finding was the fact that *Cd*-induced TTI protection is not limited to sepsis induced by infection but is also effective against the well-established zymosan challenge sepsis model. While this sepsis model reproduces the characteristic organ failure and prolonged inflammation observed with human sepsis, the zymosan dosing required to induce lethality can vary depending on the vendor source and production lot. In addition, there is a more limited lethal threshold range with the use of a single large bolus administration of an abiotic PRR ligand compared with inoculation with live replicating microbes which can increase exposure to PRR ligands over time **(** Stortz et al., 2017 **)**. Regardless of these caveats, these results showing protection against both biotic and abiotic sepsis exemplifies the role of TTI against sepsis and supports the potential of this form of TII as a vaccination strategy to mitigate sepsis.

While immunization with live low virulence *Candida* species is highly effective against sepsis, use of abiotic immunogens is a more attractive vaccine strategy. In these studies, we observed equivalent broad-spectrum protection against sepsis using abiotic fungal compounds, including modified β-glucan and d-zymosan. Protection against sepsis was also concomitant with significantly reduced *C. albicans* and *S. aureus* in most tissues (many animals had cleared the infection) at study endpoint. This is similar to protection elicited by immunization with live *C. dubliniensis* (Lilly et al., 2021). Of note, while protection was observed with various doses/timing of modified β-glucan or d-zymosan, we found that survival was most consistent with lower sepsis-associated morbidity when mice were immunized with the higher (β-glucan) or multiple (d-zymosan) dosing regimens. In contrast, neither unmodified β-glucan nor spherical β-glucan particles (WGP dispersible) induced appreciable protection against sepsis compared with alkali treated (modified) β-glucan. Alkalinization of β-glucan products including zymosan is a method used to remove TLR stimulating properties while retaining dectin-1 signaling **(** Gantner et al., 2003 **)**. This indicates that fungal cell wall ligands and PRR signaling are major inducers of TTI with strong support for a requirement of dectin-1 in this model.

Several studies have reported induction of protective trained immunity using exclusively untreated purified β-glucan in various models (*in vitro* and *in vivo*). However, the responses induced by untreated β-glucan are largely proinflammatory and associated with improved antimicrobial activity **(** Ferwerda et al., 2008 **;** Quintin et al., 2012 **;** Namakula et al., 2020 **)**. One *in vitro* study comparing monocyte responses to β-glucan, BCG, or oxLDL demonstrated that while all three can induce pro-inflammatory responses, only β-glucan could induce significant increases in anti-inflammatory cytokines (i.e., IL-10) and only at low levels when compared to TNF, IL-6 or IL-1 **(** Bekkering et al., 2016 **)**. Further, it was demonstrated that signaling *via* both dectin-1 and TLR receptors was required to induce pro-inflammatory cytokines from monocytes *in vitro* **(** Ferwerda et al., 2008 **)**. Therefore, we can speculate that removal of TLR stimulating properties may shift β-glucan responses towards anti-inflammatory cytokine production required to restrain pathological inflammation induced in sepsis models.

We have previously reported that Gr-1+ cells are required for mediating TTI protection (Lilly et al., 2018; Lilly et al., 2019; Lilly et al., 2021). Here we expand on this observation and demonstrate that for both live (IAI) and abiotic (zymosan) sepsis models, and with both live or abiotic immunization strategies, protection was completely abrogated following depletion of Gr-1+ cells. Taking into account a similar role for Gr-1+ cells in trained innate protection was reported for both i.p. or i.v. immunization with live *C. dubliniensis* against both polymicrobial IAI or monomicrobial *C. albicans* bloodstream infection (Lilly et al., 2018; Lilly et al., 2019; Lilly et al., 2021), these data provide considerable support for a primary role of Gr-1+ cells as MDSCs in protection against sepsis. Importantly, depletion with anti-Gr-1 antibodies targets both Ly6G+ and Ly6C+ cells, effectively depleting both granulocytic MDSCs (G-MDCSs) and monocytic MDSCs (M-MDSCs). Gr-1^+^ MDSCs are well-studied suppressor cells involved in the pathogenesis of sepsis (Schrijver et al., 2019) and in human fungal infections (Rieber et al., 2015); however, whether these cells are beneficial or harmful depends on a number of factors. Our observations support the concept that Gr-1^+^ MDSCs play an important role in restraining acute sepsis along with our previous studies demonstrating reductions in pro-inflammatory cytokines (Lilly et al., 2021).

In contrast to the sole protective role for Gr-1+ cells observed in the majority of studies presented here, in the case of modified β-glucan immunization there was a clear role for both Gr-1+ cells and macrophages. Monocytes/macrophages have been implicated as the primary cell population involved in TII responses, with enhanced cytokine production and improved antimicrobial activity following training with β-glucan (van der Meer et al., 2015; Moorlag S. et al., 2020). This significant role for macrophages in β-glucan-induced protection against sepsis may indicate an enhanced antimicrobial effect prior to endpoint contributing to the increased survival in mice following lethal challenge. Of note, previous work demonstrating β-glucan-induced antimicrobial activity was carried out using unmodified β-glucan (Quintin et al., 2012; Moorlag S. et al., 2020). It is possible that the differences between our β-glucan vaccinations and prior work could be the result of the alkalinization steps used herein. Likewise, the strength and quality of signaling by a live organism may differ from abiotic β-glucan recognition *in vivo*, leading to alternative immune stimulation (i.e., Gr-1+ cells vs macrophages). This does not necessarily explain why d-zymosan, which has been depleted of TLR-2 activity but not dectin-1 signaling, does not show strong roles for both innate cells in a similar manner to β-glucan; however, there was a trend toward a small role for macrophages in d-zymosan-induced protection. The overall structure and composition of both *Cd* cell wall and d-zymosan are more complex than purified β-glucan, and this may also affect the immune signaling and recognition along with the quality of host responses. We also observed similar partial phenotypes in the zymosan sepsis model. However, this was also concomitant with reduced mortality observed in unvaccinated mice. Hence, there may be a role for additional cell types that we have not investigated in this sepsis model, which will be the focus of future studies.

While *Cd*-mediated protection was maintained in both polymicrobial *Ca/Ec* IAI and abiotic zymosan-induced sepsis models, none of our immunization strategies were able to provide protection when mice were challenged with a lethal dose of LPS. This was surprising considering protective effects observed during *Ca/Ec* IAI and the fact that *E. coli* endotoxin (LPS) is the primary inducer of sepsis/mortality in *E. coli* infections (Roger et al., 2009). However, there are distinct differences in LPS sepsis compared with infection-based sepsis models. LPS-induced sepsis is considered an intoxication model, in which LPS is delivered as a single, large bolus of toxin, in contrast to the complex and repeated interactions between the microbial PAMPs and host PRRs that occur during polymicrobial IAI-mediated sepsis in mice and humans (Remick and Ward, 2005; Lewis et al., 2016). LPS administration in mice rapidly induces high circulating inflammatory cytokine levels, which peak much earlier than infection-induced sepsis (Rittirsch et al., 2007; Chen et al., 2014). Additionally, physiological effects are dose-dependent and large doses of LPS are required to induce responses similar to septic shock in mice (van der Poll, 2012; Chen et al., 2014). Therefore, while our abiotic and live immunization strategies protected against sepsis resulting from infection, these appear unable to protect against all chemical inducers of inflammation.

Overall, the results of this study have provided a better understanding of the breadth and scope of TTI protection against polymicrobial sepsis induced by *Cd* vaccination, and also identified several abiotic inducers of this TTI that are equally effective at blocking sepsis. The concept of broad protection also further validates that protective TTI is mediated by putative Gr-1+ suppressor cells (MDSCs) across multiple inducers of TTI and sepsis models. The nonspecific nature of the tolerogenic protection increases the potential as a promising vaccine strategy against sepsis of broad origin. Future studies are planned to investigate protection against virus-associated sepsis, including H1N1 influenza and respiratory syncytial virus (RSV). Preliminary studies indicate TTI inducers impart considerable protection against H1N1 influenza. Other future directions include investigating the protective mechanism, including cytokine and signaling requirements in the Gr-1+ cells, with preliminary studies supporting roles for IL-10 and CARD9 signaling. Finally, we plan to characterize the hematopoietic events in the bone marrow related to MDSC activation and associated epigenetic reprogramming following *Cd* or fungal cell wall component vaccination.

## Data Availability Statement

The original contributions presented in the study are included in the article/**Supplementary Material**. Further inquiries can be directed to the corresponding author.

## Ethics Statement

The animal study was reviewed and approved by Tulane Institutional Animal Care and Use Committee.

## Author Contributions

Conceptualization PF and MN. Methodology AH, EL, and MN. Validation AH, SR, and MN. Formal Analysis AH, PF, and MN. Resources and funding, MN. Manuscript drafting, editing and revising, AH, SER, PF, and MN. All authors contributed to the article and approved the submitted version.

## Funding

This work was supported by NIAID R01AI145096 (MN). We thank the support, in part, provided by the Louisiana Clinical and Translational Science (LA CaTS) Center, grant U54 GM104940, from the National Institutes of Health (NIH), and the LSUHSC Clinical and Translational Research Center (CTRC).

## Conflict of Interest

The authors declare that the research was conducted in the absence of any commercial or financial relationships that could be construed as a potential conflict of interest.

## Publisher’s Note

All claims expressed in this article are solely those of the authors and do not necessarily represent those of their affiliated organizations, or those of the publisher, the editors and the reviewers. Any product that may be evaluated in this article, or claim that may be made by its manufacturer, is not guaranteed or endorsed by the publisher.
