# Supplementary material for: Efficacy of Candida dubliniensis and Fungal β-Glucans in Inducing Trained Innate Immune Protection Against Inducers of Sepsis
Source: Front Cell Infect Microbiol. 2022 Jun 13;12:898030. doi: 10.3389/fcimb.2022.898030 (PMC9234138; doi:10.3389/fcimb.2022.898030)
Supplement: Supplementary file 1 [file Presentation_1.pptx]

## Slide 1
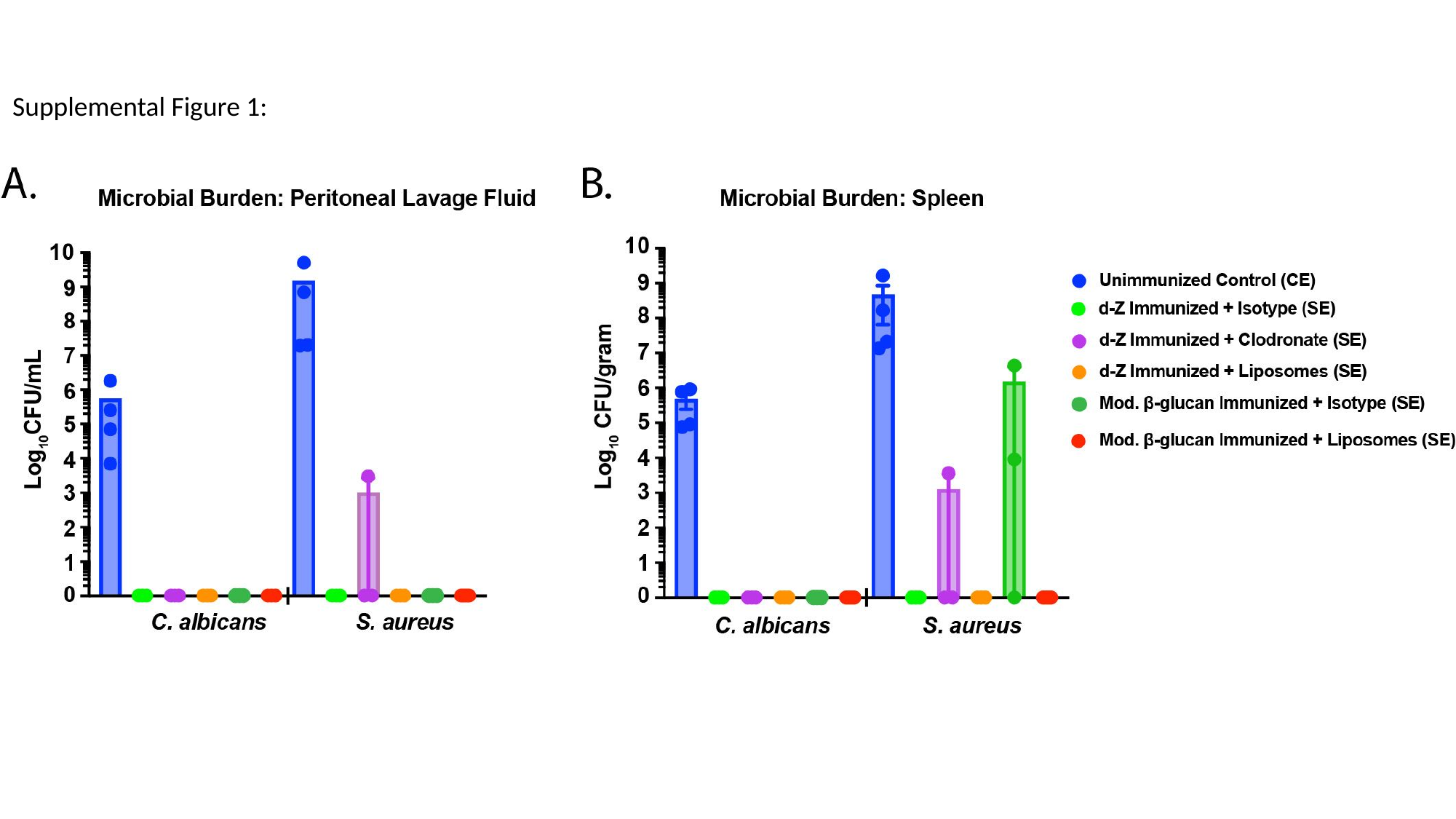

Supplemental Figure 1:

## Slide 2
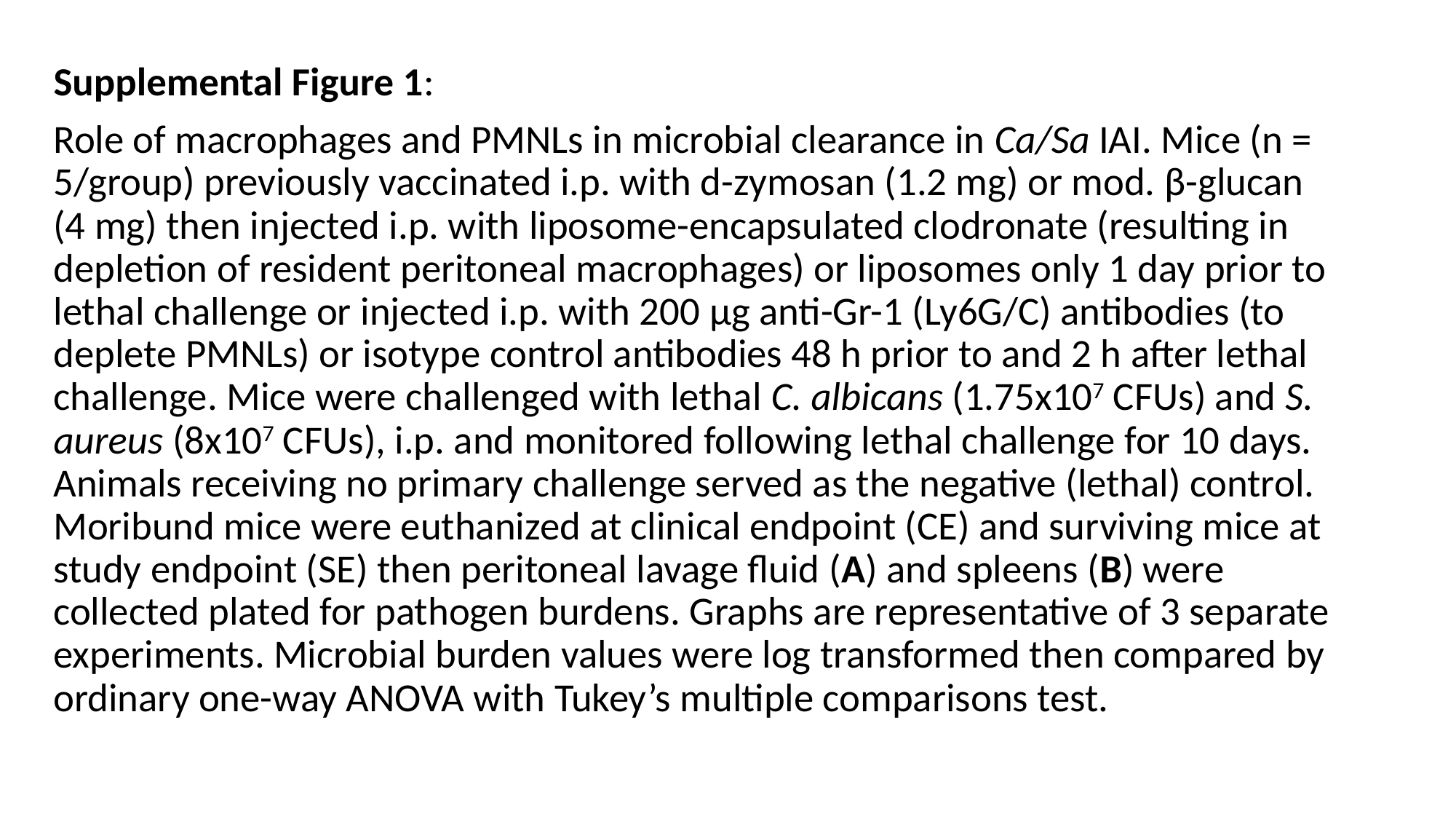

Supplemental Figure 1:
Role of macrophages and PMNLs in microbial clearance in Ca/Sa IAI. Mice (n = 5/group) previously vaccinated i.p. with d-zymosan (1.2 mg) or mod. β-glucan (4 mg) then injected i.p. with liposome-encapsulated clodronate (resulting in depletion of resident peritoneal macrophages) or liposomes only 1 day prior to lethal challenge or injected i.p. with 200 μg anti-Gr-1 (Ly6G/C) antibodies (to deplete PMNLs) or isotype control antibodies 48 h prior to and 2 h after lethal challenge. Mice were challenged with lethal C. albicans (1.75x10^7^ CFUs) and S. aureus (8x10^7^ CFUs), i.p. and monitored following lethal challenge for 10 days. Animals receiving no primary challenge served as the negative (lethal) control. Moribund mice were euthanized at clinical endpoint (CE) and surviving mice at study endpoint (SE) then peritoneal lavage fluid (A) and spleens (B) were collected plated for pathogen burdens. Graphs are representative of 3 separate experiments. Microbial burden values were log transformed then compared by ordinary one-way ANOVA with Tukey’s multiple comparisons test.
